# Relative Validity and Reproducibility of a Semi-Quantitative Food Frequency Questionnaire for Determining Nutrient Intake in Older Adults in New Zealand: The REACH Study

**DOI:** 10.3390/nu14030519

**Published:** 2022-01-25

**Authors:** Angela D. Yu, Karen D. Mumme, Cathryn A. Conlon, Pamela R. von Hurst, Nicola Gillies, Anne-Louise Heath, Jane Coad, Kathryn L. Beck

**Affiliations:** 1 College of Health, Massey University, Auckland 0632, New Zealand; angeladawnyu@gmail.com (A.D.Y.); K.Mumme@massey.ac.nz (K.D.M.); C.Conlon@massey.ac.nz (C.A.C.); P.R.vonHurst@massey.ac.nz (P.R.v.H.); 2 The Liggins Institute, University of Auckland, Auckland 1023, New Zealand; N.Gillies@auckland.ac.nz; 3 Department of Human Nutrition, University of Otago, Dunedin 9016, New Zealand; Anne-Louise.Heath@otago.ac.nz; 4 School of Food & Advanced Technology, College of Sciences, Massey University, Palmerston North 4474, New Zealand; J.Coad@massey.ac.nz

**Keywords:** nutrition assessment, food diary, food frequency questionnaire, validation, macronutrient intake, micronutrient intake, dietary assessment tool

## Abstract

Dietary intake is an important predictor of health and disease outcomes. This cross-sectional study evaluated the relative validity and reproducibility of a semi-quantitative food frequency questionnaire (FFQ) for assessing energy and nutrient intake in older adults. Dietary data were collected 2018–2019 in Auckland, New Zealand from a convenience sample of community-dwelling adults (65–74 years, *n* = 294, 37% male) using a 109-item self-administered FFQ at baseline (FFQ1) and four weeks later to assess reproducibility. FFQ1 was compared to a four-day food record to determine relative validity. Agreement between dietary assessment tools was assessed for both raw and energy-adjusted nutrient intakes using paired *t*-tests, correlation coefficients, weighted kappa statistic, Bland–Altman plots, and linear regression analysis. Energy adjustments moderately improved the relative validity and reproducibility for most nutrients. For energy and energy-adjusted nutrient intakes, the mean correlation coefficients were 0.38 (validity) and 0.65 (reproducibility); the mean weighted kappa statistics were 0.27 (validity) and 0.51 (reproducibility). A significant slope of bias was present in 54% (validity) and 25% (reproducibility) of Bland–Altman plots. The Researching Eating, Activity, and Cognitive Health (REACH) FFQ has acceptable relative validity and good reproducibility for ranking nutrient intakes in older New Zealand adults, but is less suitable for assessing absolute nutrient intakes.

## 1. Introduction

New Zealand’s population is ageing. The proportion of adults aged 65 years and over in New Zealand is expected to be more than 23% by 2043 (from 15%, 2016) [1,2]. As life expectancy increases, people are living longer but not necessarily in good health. In New Zealand, older adults carry a heavier burden of chronic diseases such as coronary heart disease, bowel cancer, and musculoskeletal disorders, with vascular disease being a primary cause of health loss [3]. Dietary intake is a key factor contributing to the onset of chronic diseases [3,4].

Energy needs decrease in later years, but the required intake of nutrients remains relatively unchanged, thus suboptimal intake of both macro and micronutrients is a concern [4,5,6]. For example, low calcium intake is commonly observed in older adults and insufficient dietary intake of calcium may contribute to bone loss and a higher risk of fractures [7,8]. Additionally, in older adults, inadequate protein intake may result in protein deficiency, causing changes in body composition, to the point that there is an increased risk of sarcopenia and muscle wasting [9,10].

As dietary intake is closely associated with the health and wellbeing of a population, it is important that dietary intake is accurately assessed [11,12]. However, assessing typical dietary intake in older adults is challenging, due to declining cognition and changes in eating habits associated with the ageing process. Dietary intakes can also be affected, for instance by poor oral health, taste changes, poor muscle strength, and declining metabolic rates, although diets and habitual behaviours are possibly more established in older adults compared to younger populations [13,14,15,16].

Traditionally, a weighed or estimated food record has been the preferred method to measure dietary intake. However, this dietary assessment method is impractical in epidemiological research involving large populations [17,18]. In comparison, a food frequency questionnaire (FFQ) is inexpensive, readily computerised, and relatively easy for participants to complete, so is useful in larger sample sizes [19]. In an FFQ, participants are presented with a list of food items and answer how frequently each food item is consumed [20]. Although an FFQ, compared with a food record, is less useful for measuring absolute dietary intakes, a validated FFQ can reflect the typical diet and relative nutrient intakes of a population and allow researchers to identify regional and local dietary risks [20,21].

FFQs are specific to the population they are developed for and should be assessed for their relative validity and reproducibility prior to use [20]. While a range of FFQs have been validated in New Zealand adults [22,23,24,25,26,27,28], a recent, valid, and reproducible FFQ to assess multiple nutrient intakes in older New Zealand adults is not available. To our best knowledge, the most recent validated FFQ study in older New Zealand adults was conducted over 25 years ago in 53 adults (54–86 years) living in Dunedin, New Zealand [26]. Food trends change over time, so FFQs need to be updated. The aim of this study was to evaluate the relative validity and reproducibility of a semi-quantitative FFQ adapted for use in older adults and its ability to determine nutrient intake in older adults living in New Zealand.

## 2. Materials and Methods

This semi-quantitative FFQ validation and reproducibility study was undertaken as part of the REACH (Researching Eating, Activity, and Cognitive Health) study at Massey University, Auckland, New Zealand. The REACH study aimed to identify the dietary patterns of older adults and their associations with cognitive function and metabolic syndrome [29]. The 109-item self-administered semi-quantitative REACH FFQ (FFQ1) was designed to assess nutrient intake, food group intake, and dietary patterns in older adults [30]. FFQ1′s ability to measure nutrient intake was assessed for validity against a four-day food record (4-DFR), and the reproducibility was assessed against a repeat administration of FFQ1 four weeks later (FFQ2). Ethical approval for the REACH study was obtained from the Massey University Human Ethics Committee Southern A, Application 17/69. Written informed consent was obtained from all participants.

### 2.1. Participants and Recruitment 

**Figure 1:**
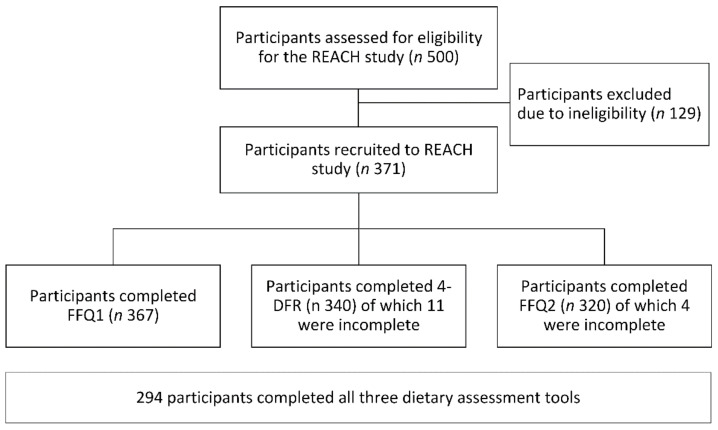
Flow chart of participants in the REACH FFQ evaluation study assessing the relative validity and reproducibility of a semi-quantitative food frequency questionnaire for assessing nutrient intake in 294 older New Zealand adults. Abbreviations: 4-DFR, four-day food record collected between the FFQ administrations; FFQ1, first semi-quantitative food frequency questionnaire; FFQ2, semi-quantitative food frequency questionnaire re-administered four weeks later; REACH, Researching Eating, Activity, and Cognitive Health.

Participants were males and females aged 65 to 74 years, living independently (i.e., not requiring assistance with daily activities or full-time care) and proficient in English. Participants were excluded if there was a previous diagnosis of dementia; they were taking medication which may affect cognitive function; or if they had or previously had any health condition that may influence cognitive function. Participants were recruited through social media, posters in public areas, radio stations, retirement villages, and by word of mouth. Participants were screened based on the eligibility criteria through telephone or email. Only one person per household was accepted. The sample size was determined by the number of REACH participants who completed three dietary assessments (FFQ1, FFQ2, and the 4-DFR). Recruitment and data collection occurred between March 2018 and May 2019. Figure 1 shows the flow of participants through the current study.

### 2.2. Development of the Semi-Quantitative FFQ

The semi-quantitative FFQ was derived from a validated New Zealand FFQ designed to measure iron-related dietary patterns in young women [31]. Changes were made to include serving sizes and food items not included in the original validation study as they were not typically related to iron nutrition, e.g., confectionary. Serving sizes were based on commonly eaten amounts guided by FOODfiles, the New Zealand food composition database [32]. Food items were combined to shorten the original FFQ from 144 to 109 food items, e.g., two seed food items became one. The FFQ was further cross-checked against the New Zealand Women’s Food Frequency Questionnaire to ensure all relevant food groups were covered [23]. The final semi-quantitative FFQ asked how often a serving of a food item was eaten in the last month. There were ten frequency response options ranging from “I never eat this food” to “six or more times per day”. A final open question invited participants to add any recently consumed foods not captured by the 109 food item questions. The frequency and portion size of these extra items were incorporated into the FFQ by the researcher at the time of analysis. Three example questions were provided at the beginning of the FFQ to demonstrate how the FFQ should be completed. The FFQ was pilot tested on ten individuals in the study age range for understanding and readability, and no changes were made to the FFQ as a result of pilot testing. The FFQ was administered online using SurveyMonkey [33]. The 109 food items and their serving size are provided in Table S1.

### 2.3. Data Collection 

During an appointment at the Human Nutrition Research Unit, Massey University, Auckland, New Zealand, demographic information (age, sex, ethnicity, education levels) were collected using a self-administered questionnaire. Anthropometric measurements were collected by trained researchers using a standardised protocol [34]. Body mass index (kg/m^2^) was calculated from height (using a stadiometer (SECA)) and weight (using Tanita electronic scales).

FFQ1, the first self-administered online FFQ, was conducted during this onsite appointment, with a researcher available during completion of FFQ1 to answer any questions. At this same appointment, participants were asked to subsequently complete a 4-DFR at home. Participants were advised to record their usual diet over four consecutive days which included at least one weekend day. Participants watched an instructional video describing how to complete the 4-DFR including instructions on describing foods (e.g., type, brand, cooking methods) and estimating food quantities. There was an opportunity to ask questions after the video and an email address was provided if the participants had further questions. To assist with estimating food quantities, supplementary pictures of food portions on a standard size plate were provided [35]. Recipes used in home-cooked items were requested to be mailed back with the completed 4-DFR via a pre-paid envelope. Where missing or incorrect information was suspected in the 4-DFR, participants were contacted by telephone or email for further clarification. Four weeks after their first appointment participants were contacted by email with a request to complete the second FFQ (identical to the first) using a link to the online survey platform [33].

### 2.4. Data Entry and Management

Missing values from the FFQs (<1% of all dietary data) were imputed using the multiple imputation chained equations method [36] with 5 imputations and 20 iterations. Predictors used were food items, age, sex, education, and living situation (alone or with others). There were no differences in characteristics between participants with and without missing data.

The 4-DFRs were entered into FoodWorks 10 [37] by trained nutritionists and dietitians using a common food assumption list to ensure quality and consistency. Final data entry checks included a single overview of entries by a New Zealand registered dietitian. The original food records were further checked for accuracy when data reports and histograms identified outliers. Any potential errors were checked back to the original food diaries. FoodWorks 10 [37] was used to summarise the mean daily intakes for total energy and macro and micronutrients. The nutrient list for analysis included total energy, protein, carbohydrate, total sugars, dietary fibre, alcohol, total fat, saturated fat, monounsaturated fat, polyunsaturated fat, cholesterol, thiamine, riboflavin, niacin, vitamins A, B6, B12, C, E, folate, beta-carotene, calcium, iron, iodine, magnesium, phosphorus, selenium, potassium, and zinc. Supplement use was excluded from the analysis. The New Zealand food composition database FOODfiles 2016 [32] was prioritised in data entry. For foods not covered by the New Zealand nutrient database, Australian food databases were used, including AusFoods 2017 or AusBrands 2017 [38]. Some foods from the 4-DFR could not be matched to foods in the FFQ. These foods were salt, pepper, other seasonings, lecithin, and artificial and natural sweeteners (all having minimal contributions to micronutrient intake).

A daily intake (g/day) for each food item in both FFQ1 and FFQ2 was calculated using the stated portion size and the frequency options. Nutrient intake from the FFQs’ food item intake was calculated using R [39] by applying a mapping file of selected nutrients to daily intake. The mapping file was based on foods from FOODfiles 2016 [32], AusFoods 2017, or AusBrands [38] that were as representative as possible of each of the 109 food items in the FFQ. For example, the FFQ food item ‘sweetened cereals’ was represented by ‘Nutri-Grain, Kellogg’s fortified’ in the FOODfiles 2016 database [32]. In some instances, a composite of foods was used, e.g., ‘salad vegetables’ was represented by ‘lettuce’, ‘celery’, and ‘cucumber’ in the FOODfiles 2016 database [32]. These decisions were made in collaboration with three members of the research team (AY, KM, KB).

Average daily energy intake was considered implausible when outside the following parameters: 2090–14,640 kJ for women and 3350–16,740 kJ for men [40]. All participants fell within these ranges and therefore no participants were excluded from the analysis.

### 2.5. Statistical Analysis

All statistical analyses were conducted with the Statistical Package for the Social Sciences (SPSS) software version 27 (IBM SPSS, Inc., Chicago, IL, USA). Firstly, data were checked for normality of distribution visually using Q–Q plots, histograms, and Kolmogorov–Smirnov and Shapiro–Wilk tests. Data were reported as means and standard deviations (SD). Analyses were conducted using both raw and energy-adjusted data where the nutrient intake was adjusted for energy intake (nutrient intake/energy intake (MJ)) [41].

To assess the relative validity of energy and nutrient intakes from the FFQ1 against those from the 4-DFR, a range of statistical methods were conducted. Pearson (or Spearman for non-normally distributed data) correlation coefficients were used to compare energy and nutrient intakes, with the magnitude of the correlation (0 to 1) indicating the strength of the relationship. Correlation coefficients of 0.50 or higher were considered a good outcome, between 0.20 and 0.49 acceptable, and less than 0.20 was a poor outcome [42]. Depending on normality, the paired *t*-test or Wilcoxon signed rank test was used to compare the mean intake differences between the FFQ1 and 4-DFR. An effect size (Cohen d) was calculated where a large effect was 0.80 or higher, medium between 0.50 and 0.79, and a small effect was between 0.20 and 0.49 [43]. Energy and nutrient intakes were grouped by tertile for cross-classification. It is recommended at least 50% of participants are correctly classified and less than 10% of participants are grossly misclassified into the opposite tertiles for each nutrient [44]. For further level of agreement, the weighted kappa statistic was used alongside cross-classification. The weighted kappa statistic was calculated based on the observed and expected percentage of agreement from the cross-classification table. Values of kappa over 0.60 indicate good agreement, between 0.20 and 0.60 acceptable, and less than 0.20 poor agreement [42]. The Bland–Altman scatterplots were used for visual investigation of level of agreement at an individual level. The difference between the two intake measurements (FFQ1- 4-DFR) for each nutrient was plotted on the vertical axis and the mean of the two intake measurements on the horizontal axis. Limits of agreement (LOA = mean difference ± 2 SD) were calculated [45]. Finally, a linear regression model was used to determine the degree of dependence of the predicting variable (or the slope of bias), in which the difference in nutrient intake was the dependent variable and mean nutrient intake was the independent variable [20]. The same statistical methods used to assess validity were also used to assess reproducibility, e.g., comparing FFQ1 and FFQ2. For all statistical tests, a *p*-value < 0.05 was considered statistically significant.

## 3. Results

### 3.1. Participant Characteristics

A total of 371 participants took part in the REACH study, with 367 participants completing FFQ1. Four weeks later, 320 participants completed FFQ2, and 340 participants completed the 4-DFR in between the administration of FFQ1 and FFQ2. Overall, 294 participants completed all three dietary assessment tools and were included in this study (Figure 1). Their characteristics are presented in Table 1.

### 3.2. Relative Validity of Energy and Nutrient Intakes Derived from the REACH FFQ

To assess validity, the raw mean energy and nutrient intakes (*n* = 29) from FFQ1 and the 4-DFR were compared (Table 2). The mean differences for 13 nutrient intakes (including energy) (45% of the 29 nutrients) were lower in FFQ1 compared with the 4-DFR, and the mean differences for nine nutrients (31%) were higher in FFQ1 compared with the 4-DFR (all *p* < 0.01). After adjusting for energy intake, the number of underestimated nutrient intakes in FFQ1 dropped to 25% (*n* = 7) but overestimated nutrient intakes in FFQ1 increased to 54% (*n* = 15) (all *p* < 0.05) (Table 2). Medium to large effect sizes (Cohen d ≥ 50) were noted for the mean differences in both raw and energy-adjusted intakes for sugars, monounsaturated fatty acids, thiamine, riboflavin, calcium, and selenium (Table 2).

Correlations for mean energy and nutrient intakes ranged from 0.12 (vitamin A) to 0.78 (alcohol) with a mean (SD) of 0.37 (0.11). All correlation coefficients were significant (all *p* < 0.05). After adjusting for energy, most correlation coefficients improved moderately, were all significant (all *p* < 0.02), and ranged from 0.14 (selenium) to 0.77 (alcohol) with a mean (SD) of 0.38 (0.13) (Table 2).

The percentage of participants correctly classified into the same tertiles ranged from 32% (vitamin E) to 68% (alcohol), with a mean (SD) of 45% (6%). Two nutrients had 50% or more participants correctly classified into the same tertiles: alcohol (68%) and niacin equivalents (54%) (Table 3). Only alcohol, carbohydrate, magnesium, and niacin equivalents had 10% or fewer participants grossly misclassified into opposite tertiles. After energy adjustment, eight nutrients had 50% or more participants correctly classified into the same tertiles and six nutrients had 10% or fewer participants grossly misclassified (Table 3).

Weighted kappa scores (κw) between FFQ1 and 4-DFR nutrient intakes ranged from 0.12 (folate) to 0.61 (alcohol) with a mean (SD) weighted kappa score of 0.25 (0.09). Eight nutrients showed poor agreement (κw < 0.20), 20 nutrients (including energy intake) had acceptable agreement (κw between 0.21–0.60), and alcohol had good agreement (κw between 0.61–0.80). The weighted kappa scores improved moderately after energy adjustment (mean (SD) = 0.27 (0.10)). Alcohol had good agreement between the methods (κw = 0.62), 22 nutrients had acceptable agreement (0.20 ≤ κw ≤ 0.60), and five nutrients had poor agreement (κw < 0.20): folate, iodine, iron, potassium, and selenium (Table 3).

Bland–Altman analysis was performed to measure the level of agreement between FFQ1 and 4-DFR and to identify outliers. For each nutrient, limits of agreement are reported in Table S2 for both raw and energy-adjusted nutrients. Most nutrients (*n* = 23, 79%) had 95% of participants within the limits of agreement, and this number reduced after the nutrients were adjusted for energy (*n* = 20, 71%) (data not shown). Six nutrients (eight energy-adjusted nutrients) had more than 5% of participants outside the limits of agreement. Alcohol (both raw and energy-adjusted) had the most at 8%.

An example of a Bland–Altman plot is provided for energy-adjusted thiamine intake (Figure S1). This plot demonstrates that as the mean intake of thiamine (between FFQ1 and 4-DFR) increases, the difference in intakes becomes greater, indicating a slope of bias (*p* < 0.001). A slope of the bias was statistically significant (*p* < 0.05) for most nutrients (*n* = 19, 66%), indicating higher intakes for these nutrients may affect the differences in intake or level of agreement between the dietary assessment tools. Results improved after energy adjustment, where the number of nutrients with a slope of bias was reduced from 19 to 15 nutrients (Table S2).

### 3.3. Reproducibility of Energy and Nutrient Intakes Derived from the REACH FFQ

To assess reproducibility, the raw mean energy and nutrient intakes (*n* = 29) from FFQ1 and FFQ2 were compared (Table S3). The mean difference for most nutrient (including energy) intakes (*n* = 26, 90%) were higher in FFQ1 compared with FFQ2 (all *p* < 0.05). The effect size for these differences was minimal to small (Cohen d > 50) (Table S3).

After adjusting for energy intake, the mean differences for four nutrient intakes were significantly higher in FFQ1 compared with FFQ2 (carbohydrate, dietary fibre, potassium, and magnesium), while the mean differences for alcohol and calcium intakes were significantly lower in FFQ1 compared with FFQ2 (*p* = 0.05) (Table S3). There was a small effect size for the mean difference in calcium intake only (Cohen d = 0.32) (Table S3).

Correlations for mean energy and nutrient intakes ranged from 0.31 (vitamin A) to 0.90 (alcohol), with a mean (SD) of 0.63 (0.10) (all *p* < 0.001). After adjusting for energy, most correlation coefficients improved moderately and ranged from 0.31 (vitamin A) to 0.90 (alcohol) with a mean (SD) of 0.65 (0.10) (all *p* < 0.001) (Table S3).

Most nutrients (including energy intake) (*n* = 28, 97%) had 50% or more participants correctly classified into the same tertiles, only vitamin B12 was the exception (47% of participants) (Table S4). All nutrients had fewer than 10% of participants grossly misclassified into opposite tertiles. After energy adjustment, all nutrients had 50% or more participants correctly classified into the same tertiles and 10% or less participants grossly misclassified (Table S4).

Weighted kappa scores (κw) between FFQ1 and FFQ2 nutrient intakes ranged from 0.39 (selenium) to 0.75 (alcohol) with a mean (SD) score of 0.52 (0.06). No nutrients showed poor agreement (κw < 0.20), 28 nutrients (including energy intake) had acceptable agreement (κw between 0.21–0.60), and alcohol had good agreement (κw between 0.61 and 0.80). After energy adjustment, the mean (SD) weighted kappa score was 0.51 (0.08), with 27 nutrients having acceptable agreement, and alcohol having good agreement (Table S4).

Using Bland–Altman analysis, 11 nutrients (38%) had 95% of participant intakes within the limits of agreement, which improved after adjustment for energy intake (*n* = 21, 75%) (Table S5). The remaining nutrients (both raw and energy-adjusted) had more than 92% of participant intakes within the limits of agreement.

A Bland–Altman plot example demonstrates the variance of difference in thiamine intake (energy-adjusted) was consistently spread across the energy-adjusted thiamine intake and no slope of bias is apparent (*p* = 0.91) (Figure S2). The slope of the bias was statistically significant (*p* < 0.05) for six nutrients (21%). After energy adjustment, seven nutrients (25%) had a significant slope of bias (Table S5).

## 4. Discussion

This validation study has demonstrated the REACH 109-item FFQ shows acceptable relative validity and good reproducibility for measuring energy and 28 nutrient intakes in older adults living in New Zealand (Table 4). The REACH FFQ was compared against a 4-DFR to assess relative validity, and a second, identical, FFQ that was re-administered four weeks later to assess reproducibility.

### 4.1. Validity of the FFQ

To determine relative validity of the REACH FFQ, agreement of energy and nutrient intakes between the dietary assessment tools were assessed using various statistical measures. Unadjusted correlation coefficients ranged from 0.12 to 0.78. The correlation coefficients for most energy-adjusted nutrient intakes fell within an acceptable coefficient range of 0.20 to 0.49 (*n* = 20, 71%) or fell within a good coefficient range of 0.50 or higher (*n* = 5, 18%), showing acceptable relative validity and the suitability for the nutrient intakes to be ranked. The correlation coefficient range in this study is similar to other FFQ validation studies in New Zealand adults, with raw coefficients for nutrients ranging from 0.11 to 0.59 [23], 0.11 to 0.74 [28], 0.18 to 0.85 [22], and 0.36 to 0.84 [24]. Results were also comparable to similar studies in older populations: 0.01–0.40 [13], 0.09–0.78 [46], 0.17–0.49 [47], 0.34–0.75 [26], 0.38–0.55 [48], 0.56–0.84 [49], and 0.71–0.99 [50]. After adjusting nutrient intakes for energy, validity correlations between the FFQ and the 4-DFR improved moderately for most nutrients in this and other studies [22,23,28,46]. Where studies, in older adults, only reported energy-adjusted nutrients, they also reported similar coefficient ranges: −0.09–0.57 [51], 0.17–0.70 [16], and 0.31–0.67 [52]. These validation studies used similar methodologies and reference methods.

Some nutrients with low correlations, such as vitamin A, are naturally rich in only a few food items (e.g., kumara, carrots, pumpkin), which can result in variability of intake across days. Longer periods of time (more than 4 days) are likely to be needed for accurate measurements of some nutrients, though there is a trade-off between accuracy and practicality when a long data collection period is used [17]. Of interest were the nutrient intakes that were highly correlated here and in other FFQ validation studies. The three most highly correlated nutrient intakes derived from the REACH FFQ were similar to those in other studies: alcohol [16,22,24,28,46,51], carbohydrate [22,26,28,49], and cholesterol [13,22,24,26].

The REACH FFQ under- (*n* = 17, 59%) and over-estimated (*n* = 12, 41%) nutrient intake when compared with the 4-DFR. Other FFQ validation studies have reported a variety of outcomes with regards to estimating intakes. With regards to New Zealand FFQs, nutrient intake has generally been overestimated for most nutrients [22,23,28], though one FFQ (compared to a three-day food record) underestimated seven of eleven nutrients in a New Zealand European cohort (40–65 years) [24]. Studies in older populations found FFQs both under- [13,47,50] and over-estimated [16,46,51] energy and nutrient intake when compared with a food diary. In other studies, the FFQs had similar nutrient estimations [16,26,48].

In this current study, cross-classification using tertiles was poor. Ideally, for good agreement of nutrient intakes, correct classification (same tertiles) should occur for 50% or more of participants and gross misclassification (opposite tertiles) in 10% or fewer participants [42,44]. Only two nutrients (7%) had correct classification in more than 50% of participants: niacin equivalents and alcohol. Only four nutrients (14%) had fewer than 10% of participants grossly misclassified: carbohydrate, alcohol, niacin equivalents, and magnesium. Other studies reported a wide range of outcomes. Ten percent or fewer of participants were grossly misclassified in 11% to 100% of nutrient intakes [16,46,50,51]. Fifty percent or more of participants were correctly classified in 0% to 100% of nutrient intakes [16,46,51], or 70% or more of participants were within one classification for 0% to 100% of nutrient intakes [26,46,51]. However, in these studies, quartiles [16,50] and quintiles [26,46,51] were used, rather than tertiles, which makes comparing studies difficult as the number of segments used in cross-classification can affect the proportion of classification. Using quintiles instead of tertiles decreases the proportion of participants who are mis-classified but also decreases the proportion of participants correctly classified. Furthermore, cross-classification can group participants with a large variation of nutrient intakes into the same tertile and participants with very similar intakes into different tertiles depending on the tertile cut-off. Several dietary assessment validation studies in older adults did not perform a cross-classification analysis [13,26,48,49,52].

While the cross-classification analysis had a poor result, the weighted kappa was calculated to overcome agreement that may have occurred by chance in the cross-classification process [44]. Weighted kappa values ranged from 0.12 to 0.61 with a mean (SD) of 0.25 (0.09), indicating acceptable validity (Table 4). These results are comparable to similar validation studies in older adults where weighted kappa values have ranged from 0.00 to 0.77 [51], 0.08 to 0.49 [46], 0.09 to 0.50 [16], 0.16 to 0.32 [47], 0.37 to 0.50 [49], and 0.78 to 0.97 [50].

The Bland–Altman plots and subsequent linear regression analysis identified many nutrients with a slope of bias, indicating that as the mean intake (e.g., the mean of FFQ1 and 4-DFR) increased, the variation in the difference between the two intakes changed. These results suggest there is a systematic difference between the results of the two dietary assessment tools [20]. Few validation studies in older adults are reported using a Bland–Altman analysis. This may be because the analysis aims to assess agreement at a group (or population) level, whereas the correlation coefficient, cross-classification, and weighted kappa statistics are assessing the suitability of ranking the nutrient intakes [42]. However, one study (n 341, 60+ years, Spanish Mediterranean population) reported homogenous dispersions above and below the mean difference in each nutrient plot suggesting consistent agreement in absolute intake between the two dietary assessment tools (FFQ and a 24 h recall) [50].

Sometimes the second administered FFQ is validated (rather than the first FFQ) against the food record [46,47,50]. In this study, using the second FFQ may improve validity because the FFQ2 covers the month the 4-DFR is recorded. However, FFQ1 was chosen to avoid any influence the completion of the 4-DFR may have on responses to the FFQ2 [20]. The use of FFQ1 to assess validity rather than FFQ2 has been reported in other studies [16,51].

Overall, the REACH FFQ demonstrated acceptable relative validity for ranking nutrient intakes when compared to the 4-DFR (Table 4). Caution is required when measuring absolute nutrient intakes as the mean differences between the FFQ1 and 4-DFR were significantly different in 76% of nutrients. In addition, Bland–Altman analysis demonstrated the accuracy of the FFQ to assess intake of several nutrients diminished at higher levels of intake.

### 4.2. Reproducibility of Nutrients from the FFQ

To determine reproducibility of the REACH FFQ, agreement of energy and nutrient intakes between FFQ1 and FFQ2 were assessed using various statistical measures. Correlation coefficients for energy-adjusted nutrient intakes were good and ranged from 0.31 to 0.90. Results were similar to reproducibility coefficient ranges in older populations: 0.30–0.91 [50], 0.42–0.87 [16], 0.47–0.62 [48], 0.50–0.70 [52], 0.51–0.60 [47], and 0.66–0.96 [46]. In this current study, both the validation and reproducibility correlation coefficients for the intake of alcohol were highly correlated. This was similarly reported in other studies [16,22,24,28,46] and a meta-analysis of reproducibility studies [53].

The percentage difference between energy and nutrient mean intakes from FFQ1 and FFQ2 ranged from −7% to 11% indicating good agreement. Cross-classification between FFQ1 and FFQ2 showed good agreement (Table 4). As recommended by the literature [42,44], at least 50% of participants were correctly classified into the same tertiles for energy and most nutrients (*n* = 28, 97%). Only vitamin B12 was outside this range with 47% of participants correctly classified. The percentage of participants grossly misclassified into opposite tertiles ranged from 1% to 6% for all nutrients. Another study, assessing reproducibility of an FFQ in older adults, found similar cross-classification results: using quartiles, at least 50% of participants were correctly classified in 48% (*n* = 12) of nutrients and less than 10% of participants were grossly misclassified in 100% (*n* = 25) of nutrients [16].

The weighted kappa statistic demonstrated acceptable agreement between the two FFQs ranging from 0.39 to 0.75 (Table 4). The weighted kappa range was similar to other studies assessing reproducibility of FFQs in older adults; 0.24–0.40 [16], 0.37–1.00 [50], and 0.46–0.86 [46].

Bland–Altman plots indicated slopes of bias in few nutrients indicating good agreement (Table 4). This was confirmed with linear regression analysis where 22 energy-adjusted nutrients (79%) demonstrated non-significant results, indicating the difference between FFQ1 and FFQ2 was not significantly dependent on the mean intake.

In this current study, FFQ1 showed good agreement of nutrient intakes when compared with FFQ2. Higher reproducibility is observed in groups with routine and well-established diets [54], particularly noted in older compared to younger populations due to habitual behaviours [13,14]. Furthermore, higher correlations are reported in self-administered FFQs and where the second FFQ is repeated within a six-month period [20,53]. Willet (2012) suggests the ideal method to assess reproducibility is an average of repeated questionnaires over several intervals [19,20,55]; however, this is not always suitable for every research design. In this current study, there was a one-month interval between FFQ administrations, and this may be considered as a limitation. However, this interval was considered short enough to minimise any change in the diet and long enough for participants to forget their initial responses to FFQ1 [20].

Adjusting nutrient intake for energy is recommended to reduce measurement error related to the reported energy intake [14]. The analyses in this study reported both raw and energy-adjusted nutrient intakes. Energy-adjusted intakes improved correlation somewhat, and a substantial improvement was noted in the mean differences of nutrient intakes between the FFQ1 and FFQ2. Here, 26 (90%) raw nutrients had significant mean differences in intakes. This reduced to six (21%) nutrients after adjusting for energy intake, indicating better reproducibility of absolute energy-adjusted values in the REACH FFQ.

### 4.3. Strengths and Limitations 

There were various strengths to the current validation study. Considering the number of challenges in assessing dietary intake and recruiting older adults, the current study was able to obtain a large sample size (*n* = 294), higher than the 100 participants recommended for validation studies [14,19,20]. All participants were cognitively healthy and as recommended, a wide range of statistical methods were used to assess validity and reproducibility. The study employed several statistical methods to assess validity and reproducibility as fewer methods may be insufficient to provide an in-depth analysis.

There are also limitations to this study. This study did not consider dietary supplement intake. A convenience sample was recruited, so may not represent the general population and participants in the study may have been more motivated to complete the dietary assessments. Participants were relatively lean compared with the New Zealand population; 13% of participants were categorised as obese (BMI > 30 kg/m^2^) compared with obesity levels in the older New Zealand population overall of 38% [56]. Additionally, the New Zealand population identifies as 70% European, 17% Māori, 8% Pacific peoples, and 15% Asian [57], whereas the majority of study participants were European (95%) meaning the FFQ should be validated prior to use in other ethnic groups.

Under-reporting is common in self-reported dietary assessments [20]. The simplest method to identify mis-reported energy intakes is to examine extreme intakes outside of a proposed energy range; the energy cut-off applied in this study was 2090–14,640 kJ (500–3500 kcal) for females, and 3350–16,740 kJ (800–4000 kcal) for males [40]. Based on this cut-off, all reported energy intakes from the FFQs and food records were within range. However, this crude method does not consider each individual profile and may not identify all under- or over-reported energy intakes.

## 5. Conclusions

In conclusion, the REACH 109-item FFQ showed acceptable relative validity and good reproducibility for energy and nutrient intakes in older New Zealand adults (65–74 years). The REACH FFQ is considered a valid dietary assessment tool for ranking nutrient intakes rather than assessing absolute intakes. The REACH FFQ can be used in future studies regarding dietary intakes in older New Zealand European adults and associations with health outcomes. Further valid and reproducible FFQs are required in older adults in other ethnic groups living in New Zealand such as Māori, Pacific Island, and Asian groups.

## Figures and Tables

**Table 1:** Characteristics of the participants who completed three dietary assessment tools and were included in the evaluation of the relative validity and reproducibility of the REACH FFQ for assessing nutrient intake in 294 New Zealand adults.

Characteristics	Mean (SD)	*n* (%)
**Age** (years)	69.8 (2.6)	294 (100%)
**Female sex**		186 (63%)
**Ethnicity**		
European/other		279 (95%)
Māori/Pacific Islander		9 (2%)
Asian		8 (3%)
**Education Status**		
No qualification/Secondary education only		68 (23%)
Post-Secondary		118 (40%)
University		108 (37%)
**BMI** (kg/m^2^)	26.1 (4.4)	294 (100%)
Underweight BMI: <18.5 kg/m^2^		2 (1%)
Normal BMI 18.5–24.9 kg/m^2^		124 (42%)
Overweight BMI: 25.0–29.9 kg/m^2^		129 (44%)
Obese BMI: ≥30.0 kg/m^2^		39 (13%)

Abbreviations: BMI, body mass index; FFQ, semi-quantitative food frequency questionnaire; REACH, Researching Eating, Activity, and Cognitive Health.

**Table 2:** Mean daily energy and nutrient intakes from the REACH FFQ1 and the 4-DFR: validation statistics for both raw ^a^ and energy-adjusted ^b^ dietary data (*n* = 294).

Nutrient	FFQ1 Daily Intake ^a^ Mean (SD)	4-DFR Daily Intake ^a^ Mean (SD)	Mean Difference ^a,c^ (95% CI)	Percentage Difference ^a,d^ (%)	Mean Difference ^e^ *p*-Value		Effect Size ^f^		Correlation Coefficients ^g^		Correlation *p*-Value	
					Raw ^a^	Adj ^b^	Raw ^a^	Adj ^b^	Raw ^a^	Adj ^b^	Raw ^a^	Adj ^b^
Energy (MJ)	7.5 (2.2)	8.1 (1.9)	−0.6 (−0.9, −0.3)	−7.3	<0.001		0.26		0.37	-	<0.001	-
Protein (g)	80.5 (24.5)	82.8 (19.9)	−2.3 (−5.1, 0.5)	−2.8	0.11	<0.001	0.09	0.22	0.41	0.52	<0.001	<0.001
Carbohydrate (g)	178.7 (60.7)	191.0 (59.3)	−12.3 (−19.2, −5.5)	−6.5	<0.001	0.47	0.21	0.04	0.50	0.58	<0.001	<0.001
Sugars (g)	113.0 (42.6)	88.8 (33.5)	24.3 (19.6, 28.9)	27.3	<0.001	<0.001	0.59	1.29	0.44	0.48	<0.001	<0.001
Dietary fibre (g)	26.2 (9.9)	28.4 (10.0)	−2.1 (−3.3, −1.0)	−7.5	<0.001	0.80	0.22	0.02	0.51	0.54	<0.001	<0.001
Alcohol (g) ^e,g^	7.7 (9.1)	10.0 (12.8)	−2.3 (−3.5, −1.1)	−23.2	0.001 ^c^	0.02 ^c^	0.22	0.14	0.78 ^e^	0.77 ^e^	<0.001	<0.001
Total fat (g)	73.3 (24.0)	80.4 (25.4)	−7.1 (−10.5, −3.7)	−8.8	<0.001	0.18	0.24	0.08	0.29	0.45	<0.001	<0.001
SFA (g)	31.6 (12.3)	29.4 (11.2)	2.1 (0.6, 3.73)	7.3	0.008	<0.001	0.16	0.55	0.31	0.42	<0.001	<0.001
MUFA (g)	23.3 (7.9)	29.2 (10.6)	−6.0 (−7.3, −4.8)	−20.6	<0.001	<0.001	0.55	0.55	0.33	0.39	<0.001	<0.001
PUFA (g)	10.2 (4.0)	13.2 (6.0)	−3.0 (−3.7, −2.4)	−23.0	<0.001	<0.001	0.51	0.47	0.35	0.48	<0.001	<0.001
Cholesterol (mg)	284.9 (134.1)	292.7 (121.7)	−7.8 (−24.0, 8.4)	−2.7	0.34	0.25	0.06	0.07	0.40	0.55	<0.001	<0.001
Thiamine (mg)	1.0 (0.4)	1.6 (0.9)	−0.5 (−0.6, −0.4)	−33.3	<0.001	<0.001	0.63	0.55	0.36	0.29	<0.001	<0.001
Riboflavin (mg)	3.0 (1.4)	2.2 (0.8)	0.8 (0.7, 1.0)	39.1	<0.001	<0.001	0.63	0.97	0.36	0.31	<0.001	<0.001
Niacin equiv. (mg)	38.1 (11.5)	37.7 (10.0)	0.3 (−0.9, 1.6)	0.9	0.60	<0.001	0.03	0.36	0.48	0.48	<0.001	<0.001
Vitamin B6 (mg)	3.0 (1.0)	2.5 (0.9)	0.4 (0.3, 0.6)	17.9	<0.001	<0.001	0.48	0.69	0.50	0.32	<0.001	<0.001
Folate (μg)	365.8 (134.2)	376.6 (139.8)	−10.8 (−30.2, 8.3)	−2.9	0.28	0.03	0.06	0.13	0.24	0.19	<0.001	<0.001
Vitamin B12 (μg)	5.2 (4.3)	4.3 (3.8)	0.9 (0.4, 1.5)	22.2	0.002	<0.001	0.18	0.25	0.18	0.22	0.002	<0.001
β-carotene (mg)	4.5 (2.2)	3.7 (2.3)	0.9 (0.6, 1.2)	23.8	<0.001	<0.001	0.36	0.50	0.45	0.44	<0.001	<0.001
Vitamin A (mg)	1.5 (1.4)	1.1 (1.0)	0.4 (0.2, 0.6)	32.9	<0.001	<0.001	0.23	0.30	0.12	0.17	<0.05	0.004
Vitamin C (mg)	133.7 (70.9)	125.2 (70.2)	8.5 (−0,2, 17.2)	6.8	0.06	<0.001	0.11	0.25	0.42	0.39	<0.001	<0.001
Vitamin E (mg)	10.2 (3.9)	11.1 (4.3)	−0.9 (−1.5, −0.4)	−8.3	0.001	0.76	0.20	0.02	0.34	0.49	<0.001	<0.001
Calcium (mg)	1193.2 (552.1)	923.7 (341.1)	269.5 (209.9, 329.2)	29.2	<0.001	<0.001	0.52	0.85	0.40	0.36	<0.001	<0.001
Iron (mg)	10.0 (3.3)	12.4 (3.9)	−2.3 (−2.8, −1.9)	−19.0	<0.001	<0.001	0.57	0.42	0.36	0.36	<0.001	<0.001
Iodine (μg)	87.0 (36.9)	97.8 (67.6)	−10.8 (−18.8, −2.8)	−11.0	0.008	0.14	0.16	0.09	0.22	0.22	<0.001	<0.001
Potassium (mg)	3965.4 (1172.3)	3644.0 (967.2)	321.4 (190.5, 452.4)	8.8	<0.001	<0.001	0.28	0.79	0.45	0.36	<0.001	<0.001
Magnesium (mg)	340.2 (100.4)	381.9 (117.3)	−41.8 (−54.9, −28.7)	−10.9	<0.001	<0.001	0.37	0.21	0.46	0.49	<0.001	<0.001
Phosphorus (mg)	1476.4 (498.0)	1516.5 (383.4)	−40.1 (−96.7, 16.5)	−2.6	0.16	<0.001	0.08	0.21	0.40	0.38	<0.001	<0.001
Selenium (μg)	47.1 (18.6)	75.1 (41.0)	−28.1 (−32.9, −23.2)	−37.4	<0.001	<0.001	0.67	0.59	0.17	0.14	0.005	0.02
Zinc (mg)	10.5 (3.4)	10.2 (2.8)	0.3 (−0.1, 0.7)	3.0	0.14	<0.001	0.09	0.40	0.40	0.26	<0.001	<0.001

^a^ Not adjusted for energy intake. ^b^ Adjusted for energy intake (nutrient intake/energy intake (MJ)) [41]. ^c^ Mean difference = FFQ1 intake − 4-DFR intake. ^d^ Mean difference % = (FFQ1 intake − 4-DFR intake)/FFQ1 intake. ^e^ The paired *t*-test or Wilcoxon signed rank test (alcohol) was used to compare the mean differences between FFQ1 and 4-DFR. ^f^ Effect size for mean difference. Cohen d (d): small effect 0.20 ≤ d < 0.50; medium effect 0.50 ≤ d < 0.80; large effect d ≤ 0.80. ^g^ Pearson or Spearman correlation (alcohol) coefficients. Outcomes: good rho ≥0.50, acceptable rho 0.20 to 0.49, or poor rho <0.20. Significant results, *p*-value < 0.05. Abbreviations: 4-DFR, four-day food records; CI, confidence interval; FFQ1, first administered semi-quantitative food frequency questionnaire; MUFA, monounsaturated fatty acid; Niacin equiv., niacin equivalents total—the sum of the percentage of niacin, preformed and niacin equivalent from tryptophan; PUFA, polyunsaturated fatty acid; REACH, Researching Eating, Activity, and Cognitive Health; SD, standard deviation; SFA, saturated fatty acid.

**Table 3:** Cross-classification ^a^ and weighted kappa ^b^ for energy and nutrient intake from the REACH FFQ1 and the 4-DFR: validation statistics for both raw ^c^ and energy-adjusted ^d^ data (*n* = 294).

Nutrient	Correctly Classified—Same Tertiles (%) ^a^		Grossly Misclassified—Opposite Tertiles (%) ^a^		Weighted Kappa Statistics ^b^	
	Raw ^c^	Adjusted ^d^	Raw ^c^	Adjusted ^d^	Raw ^c^	Adjusted ^d^
Energy	43.5		12.2		0.23	
Protein	47.6	50.0	12.2	11.2	0.27	0.31
Carbohydrate	45.9	54.4	7.8	8.2	0.30	0.39
Sugars	44.9	46.9	10.9	12.2	0.26	0.26
Dietary fibre	44.2	50.7	12.2	9.2	0.23	0.34
Alcohol	68.0	68.7	2.7	2.0	0.61	0.62
Total fat	39.1	48.0	11.2	12.6	0.15	0.27
SFA	42.5	49.0	11.2	14.3	0.19	0.26
MUFA	39.8	45.9	14.6	13.3	0.16	0.24
PUFA	46.6	50.0	13.3	7.8	0.25	0.35
Cholesterol	42.5	47.3	15.3	9.2	0.18	0.30
Thiamine	45.2	43.5	11.2	12.9	0.26	0.22
Riboflavin	45.6	47.3	12.2	11.9	0.25	0.27
Niacin equiv.	54.1	52.4	6.5	10.9	0.41	0.34
Vitamin B6	47.3	44.6	12.9	16.0	0.26	0.20
Folate	38.4	40.8	16.7	17.0	0.12	0.14
Vitamin B12	44.2	50.3	11.2	10.9	0.25	0.32
β-carotene	43.9	44.2	13.3	13.6	0.22	0.22
Vitamin A	43.2	45.6	16.0	13.6	0.18	0.23
Vitamin C	44.2	45.2	11.6	11.2	0.24	0.26
Vitamin E	31.6	49.0	14.6	7.5	0.18	0.34
Calcium	48.3	42.5	11.6	11.9	0.29	0.22
Iron	44.2	40.8	12.2	16.3	0.23	0.15
Iodine	45.6	41.2	13.6	13.9	0.23	0.18
Potassium	42.9	40.5	10.2	14.6	0.24	0.16
Magnesium	46.9	51.7	8.8	10.9	0.30	0.33
Phosphorus	42.9	42.2	11.6	11.6	0.23	0.22
Selenium	42.9	44.6	18.4	17.3	0.15	0.18
Zinc	48.0	44.2	10.5	15.6	0.30	0.20

^a^ Cross-classification (%) outcomes: good ≥50% in same tertile or ≤10% in opposite tertile; poor <50% in same tertile or >10% in opposite tertile [42]. ^b^ Weighted kappa (κw) outcomes: good κw >0.60, acceptable κw 0.20 to 0.60, or poor κw <0.20 [42]. ^c^ Not adjusted for energy intake. ^d^ Adjusted for energy intake (nutrient intake/energy intake (MJ)) [41]. Abbreviations: 4-DFR, four-day food records; FFQ1, first administered semi-quantitative food frequency questionnaire; MUFA, monounsaturated fatty acid; Niacin equiv., niacin equivalents total is the sum of the percentage of niacin, preformed and niacin equivalent from tryptophan; PUFA, polyunsaturated fatty acid; REACH, Researching Eating, Activity, and Cognitive Health; SFA, saturated fatty acid.

**Table 4:** Summary of statistical test outcomes and interpretation for validity and reproducibility of energy and nutrient intakes from the REACH FFQ ^a^.

Statistical Test	Validity ^b^		Reproducibility ^c^	
	Raw ^d^	Energy-Adjusted ^e^	Raw ^d^	Energy-Adjusted ^e^
Correlation coefficient ^f^, mean (SD)	0.37 (0.11) Acceptable	0.38 (0.13) Acceptable	0.63 (0.10) Good	0.65 (0.10) Good
Cross-classification ^g^, mean (SD)	Poor	Poor	Good	Good
% in same tertiles	45 (6)	47 (6)	61 (5)	61 (5)
% in opposite tertiles	12 (3)	12 (3)	4 (1)	4 (2)
Weighted kappa value ^h^, mean (SD)	0.25 (0.09) Acceptable	0.27 (0.10) Acceptable	0.52 (0.06) Acceptable	0.51 (0.08) Acceptable
Percentage difference ^k^	Acceptable	Acceptable	Good	Good
% difference within ±10.9%	52	43	93	96
% difference between ±11 and 20%	10	25	7	4
% difference beyond ±20%	38%	32%	0%	0%
Difference between mean intakes ^m^	76% Poor	78% Poor	90% Poor	21% Good
Bland–Altman ^n^	Poor	Poor	Good	Good
Presence of bias %	66	54	21	25

^a^*n* = 294. ^b^ FFQ1 (test) and 4-DFR (reference). ^c^ FFQ1 (test) and FFQ2 (reference). ^d^ Not adjusted for energy intake. ^e^ Adjusted for energy intake (nutrient intake/energy intake (MJ)) [41], ^f^ Pearson or Spearman correlation (alcohol) coefficients. Interpretation: good ≥0.50; acceptable 0.20–0.49; poor <0.20 [42]. ^g^ Calculated using tertiles. Interpretation: % of participants in same tertile, good ≥50%, poor: <50%; % of participants in opposite tertile, good ≤10%, poor >10% [42]. ^h^ Interpretation: good ≥0.61; acceptable 0.20–0.59; poor <0.20 [42]. ^k^ Mean difference percentage = (FFQ1 intake − reference intake)/FFQ1 intake. Interpretation: good 0–10%; acceptable 11–20%; poor >20% [42]. ^m^ Shows % of nutrients where the mean nutrient intakes were significantly different (*p* < 0.05) using paired *t*-test or Wilcoxon signed rank test. Interpretation: good *p* > 0.05; poor: *p* ≤ 0.05 [42]. ^n^ Shows % of nutrients with a slope of bias, calculated using linear regression. Interpretation: good *p* > 0.05; poor: *p* ≤ 0.05 [42].

## Data Availability

Consent was not obtained from study subjects to release data.

## Supplementary Materials

The following supporting information can be downloaded at: https://www.mdpi.com/article/10.3390/nu14030519/s1, Figure S1: Bland–Altman plot of agreement for thiamine intake (energy-adjusted) between FFQ1 and 4-DFR, Figure S2: Bland–Altman plot of agreement for thiamine intake (energy-adjusted) between FFQ1 and FFQ2, Table S1: List of the 109 food items in the REACH semi-quantitative FFQ and their serving sizes, Table S2: Bland–Altman analysis of the mean daily nutrient intake from the REACH FFQ1 and the 4-DFR (validation statistics), Table S3: Mean daily energy and nutrient intakes from the REACH FFQ1 and FFQ2 (reproducibility statistics), Table S4: Cross-classification and weighted kappa for energy and nutrient intake from the REACH FFQ1 and FFQ2 (reproducibility statistics), Table S5: Bland–Altman analysis of the mean daily nutrient intake from the REACH FFQ1 and FFQ2 (reproducibility statistics).
