# Supplementary material for: Relative Validity and Reproducibility of a Semi-Quantitative Food Frequency Questionnaire for Determining Nutrient Intake in Older Adults in New Zealand: The REACH Study
Source: Nutrients. 2022 Jan 25;14(3):519. doi: 10.3390/nu14030519 (PMC8838043; doi:10.3390/nu14030519)
Supplement: Supplementary file 1 [file nutrients-14-00519-s001.zip › nutrients-1506436-supplementary.pdf]

Supplementary Material:

Relative validity and reproducibility of a semi-quantitative food frequency questionnaire for determining nutrient intake in older adults in New Zealand: the REACH study

Supplementary Table S1: List of the 109 food items in the REACH semi-quantitative FFQ and their serving size

Food item in FFQ	Serving size
**Fruit**	
Apples, pears, nashi pears	1 medium
Banana	1 medium
Citrus fruits e.g., orange, tangelo, tangerine, mandarin, grapefruit, lemon, lime	1 medium or 2 small
Stone fruit e.g., apricots, nectarines, peaches, plums, lychees	1 medium or 2 small
Avocado	¼ avocado
Olives	4 olives
Strawberries, blackberries, cherries, blueberries, boysenberries, loganberries, cranberries, gooseberries, raspberries (fresh, frozen, canned)	½ cup
Dried fruit e.g., sultanas, raisins, currants, figs, apricots, prunes, dates	2 Tbsp
All other fruit e.g., feijoa, persimmon, tamarillo, kiwifruit, grapes, mango, melon, watermelon, pawpaw, papaya, pineapple, rhubarb	1 medium or ½ cup
**Vegetables**	
Potato e.g., boiled, mashed, baked, jacket, instant, roasted	1 medium or ½ cup
Hot potato chips, French fries, wedges	½ cup
Kumara, taro, green banana, cassava e.g., boiled, mashed, baked, roasted	1 medium or ½ cup
Carrots	1 medium or ½ cup
Other root vegetables e.g., yams, parsnip, swedes, beetroot, turnips	1 medium or ½ cup
Peas, green	½ cup
Green beans, broad beans, runner beans	½ cup
Broccoli, cauliflower, brussel sprouts, cabbage (all varieties)	½ cup
Salad vegetables e.g., lettuce, cucumber, celery, sprouts	½ cup
Green leafy vegetables e.g., spinach, silver beet, swiss chard, watercress, puha, Whitloof, chicory, kale, chard, collards, Chinese kale, Bok Choy, taro leaves (palusami)	½ cup
Tomatoes (all varieties)	1 medium or ½ cup
All other vegetables e.g., corn, pumpkin, mushrooms, capsicum, peppers, courgette, zucchini, gerkins, marrow, squash, asparagus, radish, eggplant, artichoke	½ cup
Onions, leeks, garlic	1 Tbsp

Food item in FFQ	Serving size
**Meat and chicken**	
Beef, lamb, hogget, mutton, pork, veal e.g., roast, steak, fried, chops, schnitzel, silverside, casserole, stew, stir fry, curry, BBQ, hamburger meat, mince dishes, frozen dinners	Palm size or ½ cup
Chicken, turkey or duck e.g., roast, steak, fried, steamed, BBQ, casserole, stew, stir fry, curry, mince dishes, frozen dinners	Palm size or ½ cup
Liver, kidney, other offal (including pate)	½ cup
Sausages, frankfurters, cheerios[a], hot dogs	1 medium sausage
Ham, bacon, luncheon sausage, salami, pastrami, other processed meat	2 medium slices
Corn beef (canned), boil up[b], pork bones, lamb flaps, povi masima[c]	Palm size or ½ cup
Meat pies, sausage rolls	1 meat pie or 2 sausage rolls
**Fish and seafood**	
Fish fried in batter (from fish & chips shop)	1 piece of palm size fish
Albacore tuna, salmon, sardines, herring, kahawai, swordfish, carp, dogfish, gemfish, Alfonsino, rudderfish, anchovies	Palm size or ½ cup
Mackerel, snapper, oreo, barracouta, trevally, dory, trout, eel	Palm size or ½ cup
Tuna (canned), hoki, gurnard, hake, kingfish, cod, tarakihi, groper, flounder	Palm size or ½ cup
Crumbed fish e.g., patties, cakes, fingers, nuggets	1 patty/cake or 2 fingers/nuggets
Green mussels, squid	½ cup
Shellfish e.g., cockles, kina, oysters, paua, scallops, shrimp/prawn, pipi, roe	½ cup
**Eggs, nuts, soy and legumes**	
Eggs – boiled, poached, raw	1 egg
Eggs - fried, scrambled, egg based dishes including quiche, soufflés, frittatas, omelets	1 egg
Nuts e.g., peanuts, mixed nuts, macadamias, pecan, hazelnuts, brazil nuts, walnuts, cashews, pistachios, almonds	1 Tbsp
Seeds e.g., pumpkin seeds, sunflower seeds, pinenuts, sesame seeds, tahini	1 tsp
Tofu, soybeans, tempeh	½ cup
Beans (canned or dried) e.g., black beans, butter beans, haricot beans, kidney beans, cannellini beans, refried beans, baked beans, chilli beans	½ cup
Peas and lentils e.g., chickpeas, hummus, falafels, split peas, cow peas, dahl	½ cup
Vegetarian sausages / meat, vegetarian burger patty, textured vegetable protein	1 sausage or 1 patty

Food item in FFQ	Serving size
**Cereals and grains**	
Bran based cereals, muesli, porridges – e.g., rolled oats, oat bran, oatmeal, All Bran, Sultana bran	½ cup
Weetbix, cornflakes or rice bubbles	2 weetbix or ½ cup
Sweetened cereals e.g., Nutrigrain, Fruit Loops, Honey Puffs, Frosties, Milo cereal, CocoPops	½ cup
Other breakfast cereals e.g., Special K, Light and tasty	½ cup
White rice	½ cup cooked
Brown rice	½ cup cooked
White pasta, noodles e.g., spaghetti, canned spaghetti, vermicelli, egg noodles, rice noodles, instant noodles	½ cup cooked
Whole meal pasta, noodles	½ cup cooked
Couscous, polenta, congee, Bulgur wheat, quinoa e.g., tabbouleh	½ cup cooked
Pancakes, waffles, sweet buns, scones, sweet muffins, fruit bread, croissants, doughnuts, brioche	1 serve
White bread and rolls including sliced and specialty breads such as foccacia, panini, pita, naan, chapatti, ciabatta, Turkish, English muffin, crumpets, pizza bases, wraps, tortilla's, burrito, roti, rewena bread	1 medium slice or ½ medium roll
Whole meal or wheat meal bread and rolls including sliced and specialty breads	1 medium slice or ½ medium roll
Whole grain or multi grain bread and rolls including sliced and specialty breads	1 medium slice or ½ medium roll
Crackers e.g., crisp bread, water crackers, rice cakes, cream crackers, Cruskits, Mealmates, vitawheat	2 medium crackers
**Dairy products and alternatives**	
Cheese e.g., Cheddar, Colby, Edam, Tasty, blue vein, camembert, parmesan, gouda, feta, mozzarella, brie, processed	2 slices
Cottage cheese, ricotta cheese	1 Tbsp
Cream, sour cream, cream cheese, cheese spreads	1 Tbsp
Cow's milk including milk as a drink, milk added to drinks (e.g., milky coffees), milk added to cereal	1 cup
Soy milk, coconut milk, rice milk, almond milk	1 cup
Smoothies, milk shakes (made from milk, yoghurt, ice cream), milk shakes, flavoured milk	1 cup
Milk based puddings e.g., rice pudding, custard, semolina, instant puddings, dairy food	½ cup
Yoghurt	½ cup
Ice cream	½ cup

Food item in FFQ	Serving size
**Non-alcoholic drinks**	
Hot chocolate, drinking chocolate, Cocoa, Ovaltine, Nesquik, Milo	1 cup
Coffee (all varieties)	1 cup
Tea	1 cup
Herbal tea, fruit tea	1 cup
Low calorie cordials	1 glass
Cordials including syrups, powders e.g., Raro	1 glass
Fruit and vegetable juices (all varieties)	1 glass
Sports drinks e.g., Powerade	1 glass
Energy drinks e.g., Red Bull, V	1 glass
Diet soft/fizzy drinks e.g., Sprite Zero, Diet Coke, Coke Zero	1 glass
Soft/fizzy drinks e.g., Sprite, Coke	1 glass
Water including tap, bottled or sparkling water	1 glass
**Alcohol**	
Beer, lager, cider (all varieties)	1 can or bottle
Red wine	1 small glass
White wine	1 small glass
Port, sherry, liquors	1 small glass
Spirits e.g., gin, brandy, whiskey, vodka	1 shot or 30 ml
Ready to drink alcoholic beverages	1 can or bottle

Food item in FFQ	Serving size
**Miscellaneous foods and snacks**	
Cakes, slices, pastries	1 medium serve
Non-milk based puddings e.g., pavlova, sweet pastries, fruit pies, trifle	1 medium serve
Biscuits, plain	2 biscuits
Biscuits, chocolate or cream filled	2 biscuits
Butter, ghee	1 tsp
Margarine	1 tsp
Vegetable oils	1 tsp
Sugar (all varieties) added by you to food / drinks	1 tsp
Jam, marmalade, honey, syrups, sweet spreads or preserves	1 tsp
Marmite, vegemite	1 tsp
Coconut cream	1 Tbsp
Coconut oil	1 Tbsp
Creamy dressings e.g., mayonnaise, tartar, thousand island, ranch dressing	1 Tbsp
Light dressings e.g., French and Italian dressing, balsamic vinegar	1 Tbsp
White sauce, cheese sauce, gravies	1 Tbsp
Tomato sauce, barbeque sauce, sweet chilli sauce	1 Tbsp
Pickles, chutney, mustard	1 Tbsp
Spices e.g., turmeric, ginger, cinnamon	1 tsp
Soup, homemade or canned	1 cup
Muesli or cereal bar (all varieties)	1 bar
Potato crisps	½ cup
Sweets, lollies	5-6 lollies
Chocolate (all other varieties)	4 squares

[a] processed sausage

[b] traditional Māori (indigenous people of New Zealand) food consisting of boiled meat and vegetables

[c] brined beef brisket

Abbreviations: FFQ, food frequency questionnaire; REACH, Researching Eating, Activity, and Cognitive Health

Supplementary Material:

Relative validity and reproducibility of a semi-quantitative food frequency questionnaire for determining nutrient intake in older adults in New Zealand: the REACH study

Supplementary Table S2: Bland-Altman analysis of the mean daily nutrient intake from the REACH FFQ1 and the 4-DFR: validation statistics for both raw[a] and energy adjusted[b] dietary data (*n* 294)

Nutrient	Raw[a]			Adjusted[b]		
	Mean difference[c] (LoA[d])	β- coefficient (SE)	*P*-value[e]	Mean difference[c] (LoA[d])	β- coefficient (SE)	*P*-value[e]
Energy (MJ)	-0.6 (-5.1, 3.9)	0.15 (0.08)	0.056	-	-	-
Protein (g)	-2.3 (-50.2, 45.7)	0.29 (0.07)	<0.001	0.4 (-3.3, 4.1)	-0.14 (0.07)	0.040
Carbohydrate (g)	-12.3 (-129.8, 105.1)	0.03 (0.07)	0.629	0.2 (-7.0, 7.3)	-0.14 (0.06)	0.021
Sugars (g)	24.3 (-55.8, 104.3)	0.33 (0.07)	<0.001	4.0 (-2.1, 10.0)	0.07 (0.07)	0.289
Dietary fibre (g)	-2.1 (-21.4, 17.1)	-0.01 (0.07)	0.871	0.0 (-1.8, 1.8)	0.02 (0.06)	0.806
Alcohol (g)[d]	-2.3 (-22.7, 18.1)	-0.43 (0.06)	<0.001	-0.2 (-2.7, 2.3)	-0.32 (0.06)	<0.001
Total fat (g)	-7.1 (-64.8, 50.6)	-0.09 (0.09)	0.294	-0.1 (-3.5, 3.3)	-0.32 (0.07)	<0.001
SFA (g)	2.1 (-25.0, 29.3)	0.13 (0.08)	0.114	0.5 (-1.4, 2.5)	-0.13 (0.07)	0.093
MUFA (g)	-6.0 (-27.5, 15.4)	-0.44 (0.08)	<0.001	-0.5 (-2.2, 1.3)	-0.60 (0.07)	<0.001
PUFA (g)	-3.0 (-14.6, 8.5)	-0.58 (0.08)	<0.001	-0.3 (-1.3, 0.8)	-0.51 (0.07)	<0.001
Cholesterol (mg)	-7.8 (-284.3, 268.7)	0.14 (0.08)	0.074	1.1 (-32.6, 34.9)	0.02 (0.06)	0.704
Thiamine (mg)	-0.5 (-2.1, 1.1)	-1.02 (0.07)	<0.001	-0.1 (-0.2, 0.1)	-1.31 (0.06)	<0.001
Riboflavin (mg)	0.8 (-1.8, 3.4)	0.76 (0.07)	<0.001	0.1 (-0.1, 0.4)	0.54 (0.08)	<0.001
Niacin equiv. (mg)	0.3 (-21.3, 21.9)	0.19 (0.07)	0.007	0.4 (-1.9, 2.7)	0.10 (0.07)	0.136
Vitamin B6 (mg)	0.4 (-1.4, 2.3)	0.19 (0.07)	0.004	0.1 (-0.2, 0.3)	0.11 (0.08)	0.185
Folate (μg)	-10.8 (-342.1, 320.6)	-0.07 (0.09)	0.469	2.4 (-33.8, 38.5)	-0.17 (0.10)	0.082
Vitamin B12 (μg)	0.9 (-9.1, 11.0)	0.20 (0.10)	0.041	0.2 (-1.1, 1.4)	0.08 (0.09)	0.391
β-carotene (mg)	0.9 (-3.8, 5.6)	-0.07 (0.07)	0.349	154.1 (-448.4, 756.6)	-0.12 (0.07)	0.107
Vitamin A (mg)	0.4 (-2.7, 3.5)	0.59 (0.10)	<0.001	57.7 (-324, 439.3)	0.41 (0.10)	<0.001
Vitamin C (mg)	8.5 (-140.6, 157.6)	0.01 (0.07)	0.852	2.4 (-16.7, 21.6)	0.07 (0.08)	0.396
Vitamin E (mg)	-0.9 (-10.1, 8.3)	-0.16 (0.08)	0.052	0.0 (-0.8, 0.8)	-0.21 (0.07)	0.002
Calcium (mg)	269.5 (-748.3, 1287.4)	0.66 (0.07)	<0.001	41.3 (-53.7, 136.2)	0.50 (0.08)	<0.001
Iron (mg)	-2.3 (-10.4, 5.7)	-0.25 (0.08)	0.002	-0.2 (-1.0, 0.6)	-0.09 (0.09)	0.319

Nutrient	Raw[a]			Adjusted[b]		
	Mean difference[c] (LoA[d])	β- coefficient (SE)	*P*-value[e]	Mean difference[c] (LoA[d])	β- coefficient (SE)	*P*-value[e]
Iodine (μg)	-10.8 (-146.8, 125.2)	-0.91 (0.08)	<0.001	-0.7 (-15.3, 14.0)	-1.04 (0.08)	<0.001
Potassium (mg)	321.4 (-1914.5, 2557.4)	0.26 (0.07)	<0.001	76.9 (-114.1, 267.9)	-0.08 (0.08)	0.312
Magnesium (mg)	-41.8 (-265.3, 181.7)	-0.21 (0.07)	0.003	-1.8 (-19.0, 15.3)	-0.67 (0.06)	<0.001
Phosphorus (mg)	-40.1 (-1006.2, 925.9)	0.37 (0.07)	<0.001	6.9 (-56.8, 70.5)	0.07 (0.08)	0.376
Selenium (μg)	-28.1 (-110.7, 54.6)	-1.17 (0.08)	<0.001	-3.1 (-13.2, 7.1)	-1.16 (0.08)	<0.001
Zinc (mg)	0.3 (-6.4, 7.0)	0.25 (0.08)	0.001	0.1 (-0.5, 0.8)	0.21 (0.09)	0.020

[a] Not adjusted for energy intake.

[b] Nutrient intake is adjusted for energy intake (nutrient intake/energy intake [MJ]) [42], therefore the mean difference is expressed as intake unit per MJ.

[c] Mean difference = FFQ1 intake − 4-DFR intake.

[d] LoA being 1.96 SD of the mean difference.

[e] Simple linear regression determined if slope of bias was present. A significant result indicates bias *P*-value<0.05.

Abbreviations: 4-DFR, four-day food records; CI, confidence interval; FFQ1, first administered semi-quantitative food frequency questionnaire; LoA, limits of agreement; MUFA, monounsaturated fatty acid; Niacin equiv., niacin equivalents total - the sum of the percentage of niacin, preformed and niacin equivalent from tryptophan; PUFA, polyunsaturated fatty acid; REACH, Researching Eating, Activity, and Cognitive Health; SE, standard error; SFA, saturated fatty acid.

Supplementary Material:

Relative validity and reproducibility of a semi-quantitative food frequency questionnaire for determining nutrient intake in older adults in New Zealand: the REACH study

Supplementary Table S3: Mean daily energy and nutrient intakes from the REACH FFQ1 and FFQ2: reproducibility statistics for both raw[a] and energy adjusted[b] dietary data (*n* 294)

Nutrient	FFQ1 daily intake[a] mean (SD)	FFQ2 daily intake[a] mean (SD)	Mean difference[a,c] (95% CI)	Percentage difference[a,d] (%)	Mean difference[e] P-value		Effect size[f]		Correlation coefficients[g]		Correlation P-value	
					Raw[a]	Adj[b]	Raw[a]	Adj[b]	Raw[a]	Adj[b]	Raw[a]	Adj[b]
Energy (MJ)	7.5 (2.2)	7.1(2.1)	0.4 (2.1, 6.1)	5.8	<0.001		0.24		0.66	-	<0.001	-
Protein (g)	80.5 (24.5)	75.8 (23.0)	4.7 (2.2, 7.2)	6.2	<0.001	0.984	0.21	0.00	0.58	0.62	<0.001	<0.001
Carbohydrate (g)	178.7 (60.7)	166.6 (57.0)	12.1 (6.9, 17.3)	7.3	<0.001	0.038	0.27	0.12	0.70	0.76	<0.001	<0.001
Sugars (g)	113.0 (42.6)	105.2 (39.4)	7.8 (4.0, 11.6)	7.4	<0.001	0.215	0.23	0.07	0.67	0.67	<0.001	<0.001
Dietary fibre (g)	26.2 (9.9)	24.0 (9.2)	2.2 (1.4, -3.0)	9.2	<0.001	0.041	0.31	0.12	0.72	0.76	<0.001	<0.001
Alcohol (g)[e,g]	7.7 (9.1)	8.2 (11.8)	-0.6 (-1.5, 0.3)	-7.1	0.21	0.027	0.07	0.13	0.90	0.90	<0.001	<0.001
Total fat (g)	73.3 (24.0)	69.8 (24.9)	3.5 (1.2, 5.7)	5.0	0.002	0.429	0.18	0.05	0.68	0.75	<0.001	<0.001
SFA (g)	31.6 (12.3)	30.1 (12.6)	1.5 (0.3, 2.6)	4.9	0.010	0.458	0.15	0.04	0.69	0.72	<0.001	<0.001
MUFA (g)	23.2 (7.9)	22.1 (8.0)	1.1 (0.3, 1.9)	4.9	0.006	0.728	0.16	0.02	0.64	0.65	<0.001	<0.001
PUFA (g)	10.2 (4.0)	9.7 (4.2)	0.5 (0.1, 0.8)	4.7	0.012	0.669	0.15	0.03	0.72	0.71	<0.001	<0.001
Cholesterol (mg)	284.9 (134.1)	276.5 (122.2)	8.5 (-5.4, 22.3)	3.1	0.23	0.398	0.07	0.05	0.56	0.67	<0.001	<0.001
Thiamine (mg)	1.0 (0.4)	1.0 (0.4)	0.1 (0.0, 0.1)	8.0	<0.001	0.296	0.25	0.06	0.68	0.60	<0.001	<0.001
Riboflavin (mg)	3.0 (1.4)	2.7 (1.3)	0.2 (0.1, 0.4)	8.7	<0.001	0.110	0.22	0.09	0.65	0.67	<0.001	<0.001
Niacin equiv. (mg)	38.1 (11.5)	36.0 (11.2)	2.1 (1.1, 3.1)	5.8	<0.001	0.968	0.23	0.00	0.69	0.71	<0.001	<0.001
Vitamin B6 (mg)	3.0 (1.0)	2.8 (0.94)	0.2 (0.1, 0.2)	5.8	<0.001	0.746	0.24	0.02	0.76	0.72	<0.001	<0.001
Folate (μg)	365.8 (134.2)	337.0 (123.3)	28.8 (15.2, 42.4)	8.6	<0.001	0.121	0.24	0.09	0.58	0.60	<0.001	<0.001
Vitamin B12 (μg)	5.2 (4.3)	4.7 (2.8)	0.5 (0.1, 1.0)	11.1	0.028	0.313	0.13	0.06	0.40	0.40	<0.001	<0.001
β-carotene (mg)	4.5 (2.2)	4.2 (2.2)	0.4 (0.2, 0.6)	8.8	<0.001	0.641	0.21	0.03	0.69	0.65	<0.001	<0.001
Vitamin A (mg)	1.5 (1.4)	1.3 (0.8)	0.2 (0.0, 0.3)	13.6	0.026	0.293	0.13	0.06	0.31	0.31	<0.001	<0.001
Vitamin C (mg)	133.7 (70.9)	123.0 (70.6)	10.7 (4.0, 17.4)	8.7	0.002	0.320	0.18	0.06	0.66	0.65	<0.001	<0.001

Nutrient	FFQ1 daily intake[a] mean (SD)	FFQ2 daily intake[a] mean (SD)	Mean difference[a,c] (95% CI)	Percentage difference[a,d] (%)	Mean difference[e] P-value		Effect size[f]		Correlation coefficients[g]		Correlation P-value	
					Raw[a]	Adj[b]	Raw[a]	Adj[b]	Raw[a]	Adj[b]	Raw[a]	Adj[b]
Vitamin E (mg)	10.2 (3.9)	9.6 (3.7)	0.6 (0.3, 0.9)	6.5	<0.001	0.704	0.22	0.02	0.72	0.76	<0.001	<0.001
Calcium (mg)	1193.2 (552.1)	1102.2 (508.4)	91.0 (39.6, 142.3)	8.3	0.001	<0.001	0.20	0.32	0.65	0.79	<0.001	<0.001
Iron (mg)	10.0 (3.3)	9.3 (3.2)	0.7 (0.4, 1.0)	7.3	<0.001	0.206	0.24	0.07	0.61	0.58	<0.001	<0.001
Iodine (μg)	87.0 (36.9)	82.0 (35.7)	5.1 (1.4, 8.7)	6.2	0.006	0.560	0.16	0.03	0.62	0.59	<0.001	<0.001
Potassium (mg)	3965.4 (1172.3)	3680.7 (1108.3)	284.7 (174.7, 394.8)	7.7	<0.001	0.033	0.30	0.13	0.65	0.64	<0.001	<0.001
Magnesium (mg)	340.2 (100.4)	317.0 (94.6)	23.2 (14.2, 32.1)	7.3	<0.001	0.032	0.30	0.13	0.68	0.70	<0.001	<0.001
Phosphorus (mg)	1476.3 (498.0)	1374.9 (458.9)	101.4 (55.5, 147.4)	7.4	<0.001	0.109	0.25	0.09	0.65	0.68	<0.001	<0.001
Selenium (μg)	47.1 (18.6)	45.6 (20.8)	1.5 (-0.8, 3.8)	3.3	0.205	0.210	0.07	0.07	0.48	0.62	<0.001	<0.001
Zinc (mg)	10.5 (3.4)	9.8 (3.2)	0.6 (0.27, 0.98)	6.4	0.001	0.948	0.20	0.00	0.57	0.59	<0.001	<0.001

[a] Not adjusted for energy intake.

[b] Adjusted for energy intake (nutrient intake/energy intake [MJ]) [42].

[c] Mean difference = FFQ1 intake – FFQ2 intake.

[d] Mean difference % = (FFQ1 intake – FFQ2 intake) / FFQ1 intake

[e] The paired t-test or Wilcoxon signed rank test (alcohol) was used to compare the mean differences between FFQ1 and FFQ2.

[f] Effect size for mean difference. Cohen d (d): small effect 0.20≤d<0.50; medium effect 0.50≤d<0.80; large effect d ≤0.80.

[g] Pearson or Spearman correlation (alcohol) coefficients. Outcomes: good rho ≥0.50, acceptable rho 0.20 to 0.49, or poor rho <0.20.

Significant results, *P*-value<0.05.

Abbreviations: CI, confidence interval; FFQ1, first administered semi-quantitative food frequency questionnaire; FFQ2, second administered semi-quantitative food frequency questionnaire 1 month later; MUFA, monounsaturated fatty acid; Niacin equiv., niacin equivalents total - the sum of the percentage of niacin, preformed and niacin equivalent from tryptophan; PUFA, polyunsaturated fatty acid; REACH, Researching Eating, Activity, and Cognitive Health; SD, standard deviation; SFA, saturated fatty acid.

Supplementary Material:

Relative validity and reproducibility of a semi-quantitative food frequency questionnaire for determining nutrient intake in older adults in New Zealand: the REACH study

Supplementary Table S4: Cross-classification[a] and weighted kappa[b] for energy and nutrient intake from the REACH FFQ1 and FFQ2: reproducibility statistics for both raw[c] and energy adjusted[d] data (*n* 294)

Nutrient	Correctly classified - same tertiles (%)[a]		Grossly misclassified - opposite tertiles (%)[a]		Weighted kappa statistic[b]	
	Raw[c]	Adjusted[d]	Raw[c]	Adjusted[d]	Raw[c]	Adjusted[d]
Energy	58.8	-		-	0.49	-
Protein	59.5	54.8	5.1	7.8	0.49	0.40
Carbohydrate	63.6	65.0	3.7	2.4	0.55	0.58
Sugars	61.6	62.2	3.1	6.5	0.53	0.50
Dietary fibre	56.8	65.3	3.7	3.4	0.47	0.57
Alcohol	79.6	77.9	1.4	0.3	0.75	0.75
Total fat	61.6	61.6	3.7	3.7	0.52	0.52
SFA	61.9	60.9	3.4	4.4	0.52	0.51
MUFA	57.5	59.5	3.7	5.8	0.48	0.48
PUFA	62.9	65.6	5.1	3.7	0.52	0.57
Cholesterol	58.2	57.8	5.1	6.8	0.47	0.45
Thiamine	59.2	57.1	4.7	4.1	0.49	0.47
Riboflavin	63.3	58.5	4.1	5.4	0.54	0.47
Niacin equiv.	66.0	63.6	4.1	1.7	0.57	0.57
Vitamin B6	65.3	62.6	2.7	2.0	0.58	0.56
Folate	58.8	57.1	4.7	4.8	0.48	0.46
Vitamin B12	46.9	62.9	4.7	3.1	0.55	0.55
β-carotene	59.9	61.2	2.7	3.4	0.52	0.52
Vitamin A	59.2	56.5	6.1	7.5	0.47	0.43
Vitamin C	65.6	60.2	4.4	3.1	0.56	0.52
Vitamin E	63.3	61.6	6.1	5.1	0.53	0.51
Calcium	62.6	70.0	5.4	0.7	0.52	0.66
Iron	61.2	57.5	6.1	5.1	0.49	0.46
Iodine	60.9	53.7	3.7	7.5	0.52	0.39
Potassium	59.2	53.1	4.7	4.1	0.49	0.43
Magnesium	61.2	56.1	4.1	3.7	0.52	0.46
Phosphorus	62.9	60.2	4.4	7.1	0.53	0.47
Selenium	52.0	57.1	5.8	6.1	0.39	0.45
Zinc	56.5	58.2	4.7	5.1	0.46	0.47

[a] Cross-classification (%) outcomes: good, ≥50% in same tertile or ≤10% in opposite tertile; poor, <50% in same tertile or >10% in opposite tertile [43].
[b] Weighted kappa (κw) outcomes: good κw >0.60, acceptable κw 0.20 to 0.60, or poor κw <0.20 [43].
[c] Not adjusted for energy intake
[d] Adjusted for energy intake (nutrient intake/energy intake [MJ]) [42].

Abbreviations: FFQ1, first administered semi-quantitative food frequency questionnaire; FFQ2, second administered semi-quantitative food frequency questionnaire administered one month later; MUFA, monounsaturated fatty acid; Niacin equiv., niacin equivalents total, is the sum of the percentage of niacin, preformed and niacin equivalent from tryptophan; PUFA, polyunsaturated fatty acid; REACH, Researching Eating, Activity, and Cognitive Health; SFA, saturated fatty acid.

nutrients

MDPI

Supplementary Material:

Relative validity and reproducibility of a semi-quantitative food frequency questionnaire for determining nutrient intake in older adults in New Zealand: the REACH study

Supplementary Table S5: Bland-Altman analysis of the mean daily nutrient intake from the REACH FFQ1 and FFQ2: reproducibility statistics for both raw[a] and energy adjusted[b] dietary data (*n* 294)

Nutrient	Raw[a]			Adjusted[b]		
	Mean difference[c] (LoA[d])	β- coefficient (SE)	*P*-value[e]	Mean difference[c] (LoA[d])	β- coefficient (SE)	*P*-value[e]
Energy (MJ)	0.4 (-3.0, 3.8)	0.02 (0.05)	0.759	-	-	-
Protein (g)	4.7 (-38.3, 47.6)	0.08 (0.06)	0.175	0.0 (-3.0, 3.0)	0.08 (0.06)	0.165
Carbohydrate (g)	12.1 (-77.4, 101.6)	0.07 (0.05)	0.130	0.3 (-4.6, 5.2)	0.03 (0.04)	0.445
Sugars (g)	7.8 (-57.5, 73.1)	0.09 (0.05)	0.072	0.2 (-4.8, 5.2)	-0.03 (0.05)	0.518
Dietary fibre (g)	2.2 (-11.8, 16.2)	0.09 (0.05)	0.064	0.1 (-1.3, 1.5)	-0.07 (0.04)	0.099
Alcohol (g)[d]	-0.6 (-16.3, 15.1)	-0.3 (0.04)	<0.001	-0.1 (-2.2, 2.0)	-0.30 (0.04)	<0.001
Total fat (g)	3.5 (-34.7, 41.6)	-0.04 (0.05)	0.398	0.0 (-2.1, 2.0)	-0.02 (0.04)	0.701
SFA (g)	1.5 (-17.6, 20.6)	-0.03 (0.05)	0.512	0.0 (-1.3, 1.3)	-0.05 (0.05)	0.264
MUFA (g)	1.1 (-12.2, 14.4)	-0.02 (0.06)	0.668	0.0 (-1.0, 1.0)	0.04 (0.05)	0.468
PUFA (g)	0.5 (-5.5, 6.5)	-0.06 (0.05)	0.174	0.0 (-0.6, 0.6)	-0.11 (0.05)	0.025
Cholesterol (mg)	8.5 (-227.8, 244.7)	0.12 (0.06)	0.056	-0.7 (-27.8, 26.5)	0.28 (0.05)	<0.001
Thiamine (mg)	0.1 (-0.5, 0.7)	0.11 (0.05)	0.026	0.0 (-0.1, 0.1)	-0.03 (0.06)	0.591
Riboflavin (mg)	0.2 (-1.9, 2.4)	0.11 (0.05)	0.039	0.0 (-0.2, 0.2)	0.02 (0.05)	0.668
Niacin equiv. (mg)	2.1 (-15.4, 19.6)	0.03 (0.05)	0.535	0.0 (-1.8, 1.8)	0.01 (0.05)	0.775
Vitamin B6 (mg)	0.2 (-1.2, 1.5)	0.06 (0.04)	0.166	0.0 (-0.2, 0.2)	0.00 (0.05)	0.984
Folate (µg)	28.8 (-202.7, 260.3)	0.11 (0.06)	0.077	1.1 (-22.7, 24.9)	0.02 (0.06)	0.726
Vitamin B12 (µg)	0.5 (-7.4, 8.4)	0.58 (0.07)	<0.001	0.0 (-0.9, 1)	0.45 (0.07)	<0.001
β-carotene (mg)	0.4 (-3.1, 3.8)	-0.01 (0.05)	0.874	6.9 (-491.3, 505.1)	-0.19 (0.05)	<0.001
Vitamin A (mg)	0.2 (-2.5, 2.9)	0.76 (0.08)	<0.001	10.6 (-326.4, 347.5)	0.54 (0.08)	<0.001
Vitamin C (mg)	10.7 (-103.8, 125.3)	0.01 (0.05)	0.924	0.4 (-14.6, 15.5)	-0.01 (0.05)	0.819
Vitamin E (mg)	0.6 (-4.9, 6.2)	0.05 (0.05)	0.301	0.0 (-0.5, 0.5)	-0.04 (0.04)	0.345
Calcium (mg)	91.0 (-786.3, 968.2)	0.1 (0.05)	0.065	-15.4 (-109.7, 78.8)	-0.49 (0.04)	<0.001
Iron (mg)	0.7 (-4.9, 6.3)	0.04 (0.06)	0.465	0.0 (-0.6, 0.6)	0.04 (0.06)	0.465
Iodine (µg)	5.1 (-56.7, 66.8)	0.04 (0.06)	0.489	0.1 (-6.1, 6.3)	0.1 (0.06)	0.077
Potassium (mg)	284.7 (-1593.7, 2163.2)	0.07 (0.05)	0.209	8.8 (-129.7, 147.4)	0.01 (0.05)	0.859
Magnesium (mg)	23.2 (-129.8, 176.2)	0.07 (0.05)	0.169	0.6 (-8.4, 9.6)	-0.02 (0.05)	0.739

Nutrient	Raw[a]			Adjusted[b]		
	Mean difference[c] (LoA[d])	β- coefficient (SE)	*P*-value[e]	Mean difference[c] (LoA[d])	β- coefficient (SE)	*P*-value[e]
Phosphorus (mg)	101.4, (-683.1, 886.0)	0.1 (0.05)	0.066	2.2 (-44.1, 48.6)	0.01 (0.05)	0.876
Selenium (μg)	1.5 (-38.1, 41.1)	-0.15 (0.07)	0.033	-0.1 (-4.1, 3.8)	0.02 (0.06)	0.676
Zinc (mg)	0.6 (-5.4, 6.7)	0.05 (0.06)	0.370	0.0 (-0.5, 0.5)	-0.03 (0.06)	0.594

[a] Not adjusted for energy intake.

[b] Adjusted for energy intake (nutrient intake/energy intake [MJ]) [42], therefore the mean difference is expressed as intake unit per MJ.

[c] Mean difference = FFQ1 intake – FFQ2 intake.

[d] LoA being 1.96 SD of the mean difference.

[e] Simple linear regression determined if slope of bias was present. A significant result indicates bias *P*-value<0.05.

Abbreviations: CI, confidence interval; FFQ1, first administered semi-quantitative food frequency questionnaire; FFQ2, second administered semi-quantitative food frequency questionnaire 1 month later; LoA, limits of agreement; MUFA, monounsaturated fatty acid; Niacin equiv., niacin equivalents total - the sum of the percentage of niacin, preformed and niacin equivalent from tryptophan; PUFA, polyunsaturated fatty acid; REACH, Researching Eating, Activity, and Cognitive Health; SE, standard error; SFA, saturated fatty acid.

Supplementary figures

Relative validity and reproducibility of a semi-quantitative food frequency questionnaire for determining nutrient intake in older adults in New Zealand: the REACH study

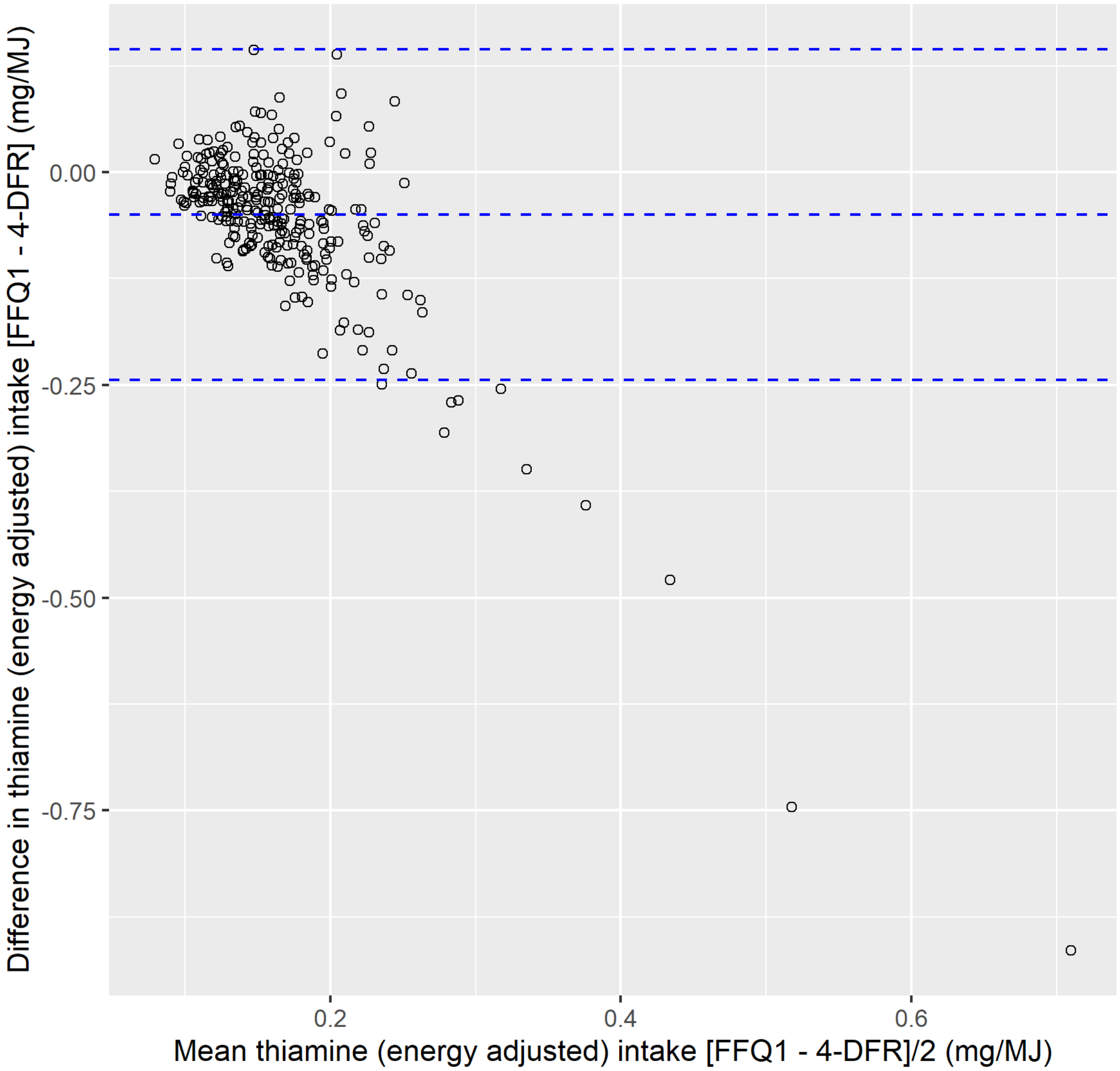

Supplementary Figure S1: The Bland-Altman plot of agreement for thiamine intake (energy adjusted) between FFQ1 and 4-DFR. The middle line represents the mean difference between two dietary assessment methods (0.05 mg/MJ); the outside dotted lines represent the limits of agreement (LOA = mean difference ± 1.96 standard deviation). This plot shows significant slope of bias (*P*<0.001).

Supplementary figures

Relative validity and reproducibility of a semi-quantitative food frequency questionnaire for determining nutrient intake in older adults in New Zealand: the REACH study

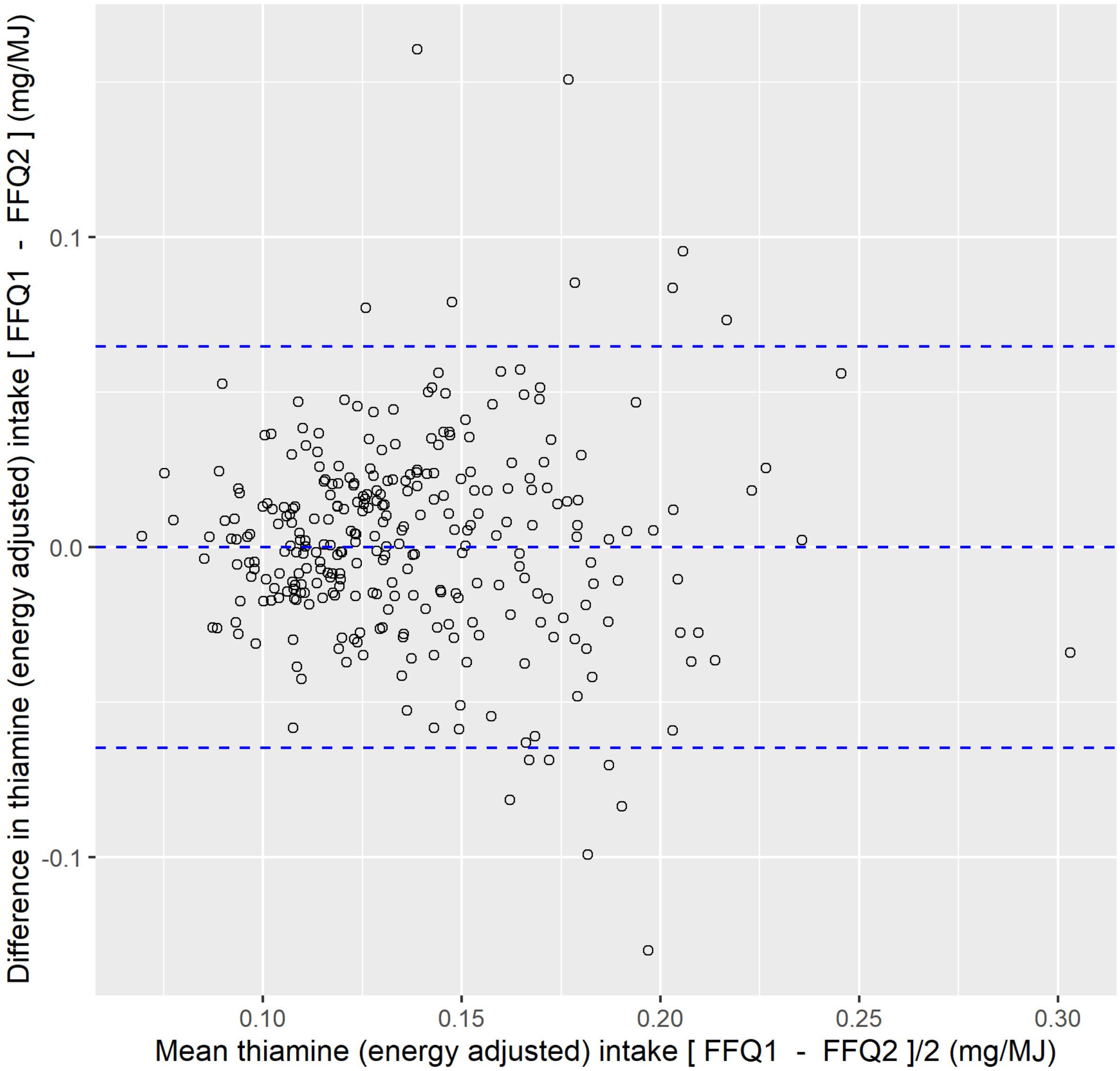

Supplementary Figure S2: The Bland-Altman plot of agreement for thiamine intake (energy adjusted) between FFQ1 and FFQ2. The middle line represents the mean difference between two dietary assessment methods (0.00 mg/MJ); the outside dotted lines represent the limits of agreement (LOA = mean difference ± 1.96 standard deviation). This plot shows no slope of bias (*P*=0.91).
